# Plant-Based Indole Alkaloids: A Comprehensive Overview from a Pharmacological Perspective

**DOI:** 10.3390/molecules26082297

**Published:** 2021-04-15

**Authors:** Faisal Omar, Abu Montakim Tareq, Ali M. Alqahtani, Kuldeep Dhama, Mohammed Abu Sayeed, Talha Bin Emran, Jesus Simal-Gandara

**Affiliations:** 1 Department of Pharmacy, International Islamic University Chittagong, Chittagong 4318, Bangladesh; faisal27.omar@gmail.com (F.O.); montakim0.abu@gmail.com (A.M.T.); 2 Department of Pharmacology, College of Pharmacy, King Khalid University, Abha 62529, Saudi Arabia; amsfr@kku.edu.sa; 3 Division of Pathology, ICAR-Indian Veterinary Research Institute, Izatnagar, Bareilly 243122, Uttar Pradesh, India; kdhama@rediffmail.com; 4 Department of Pharmacy, BGC Trust University Bangladesh, Chittagong 4381, Bangladesh; 5 Nutrition and Bromatology Group, Department of Analytical and Food Chemistry, Faculty of Food Science and Technology, University of Vigo—Ourense Campus, E32004 Ourense, Spain

**Keywords:** indole, alkaloids, pharmacological activity, pharmacokinetic profile, scientific databases

## Abstract

Plant-based indole alkaloids are very rich in pharmacological activities, and the indole nucleus is considered to contribute greatly to these activities. This review’s fundamental objective is to summarize the pharmacological potential of indole alkaloids that have been derived from plants and provide a detailed evaluation of their established pharmacological activities, which may contribute to identifying new lead compounds. The study was performed by searching various scientific databases, including Springer, Elsevier, ACS Publications, Taylor and Francis, Thieme, Wiley Online Library, ProQuest, MDPI, and online scientific books. A total of 100 indole compounds were identified and reviewed. The most active compounds possessed a variety of pharmacological activities, including anticancer, antibacterial, antiviral, antimalarial, antifungal, anti-inflammatory, antidepressant, analgesic, hypotensive, anticholinesterase, antiplatelet, antidiarrheal, spasmolytic, antileishmanial, lipid-lowering, antimycobacterial, and antidiabetic activities. Although some compounds have potent activity, some only have mild-to-moderate activity. The pharmacokinetic profiles of some of the identified compounds, such as brucine, mitragynine, 7-hydroxymitragynine, vindoline, and harmane, were also reviewed. Most of these compounds showed promising pharmacological activity. An in-depth pharmacological evaluation of these compounds should be performed to determine whether any of these indoles may serve as new leads.

## 1. Introduction

**Figure 1:**
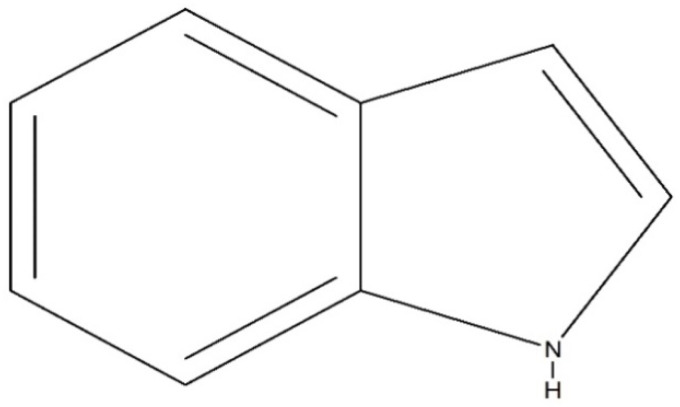
Chemical structure of indole.

Nature has always been a blessing for the field of medicine, and peoples throughout history have used natural substances for the treatment of various diseases. The sources of natural substances can be both plants and animals, and an enormous number of pharmacologically active compounds have been derived from natural sources. Many compounds isolated from natural sources have been used as drugs for treatment purposes, either with or without modifications. Through the work of ongoing research, thousands of active compounds have been isolated from natural sources, which can be classified into multiple compound classes. Alkaloids refer to a broad class of compounds, and many of the isolated bioactive compounds have been further classified as indole alkaloids. Many of the therapeutically active indole alkaloids (Figure 1) are isolated from plants, and these compounds have had a noticeable impact on the practice of medicine. Adolf von Baeyer was the first to synthesize indole from oxindole using zinc dust in 1866 [1]. Due to the occurrence of adverse effects following treatment with existing drug molecules, the search for new compounds associated with fewer adverse effects has gained immense attention from medicinal chemists and other scientists worldwide. Some of the indole compounds that have since been developed, including vincristine and vinblastine (anticancer agents), reserpine (an antihypertensive agent), physostigmine (a cholinesterase inhibitor), and ajmaline (an anti-arrhythmic agent), are now used as therapeutic drugs.

This review summarizes current insights regarding the development of plant-based indole alkaloids with biological activities. In this review, 100 indole alkaloids are discussed, including an overview of their pharmacological activities and pharmacokinetic profiles. All information on the compounds was retrieved from scientific databases, such as Springer, Elsevier, ACS Publications, Taylor and Francis, Thieme, Wiley Online Library, ProQuest, MDPI, and online scientific books.

## 2. Indole Alkaloids: An Overview

Indole (C_8_H_7_N) is a weakly basic molecule consisting of a pyrrole ring fused to a benzene nucleus, and ten π electrons move throughout the structure. The basic environment of indole alkaloids is thought to be caused by the delocalization of the lone pair of nitrogen electrons into the free circulation of the π electronic system. This results in indole becoming protonated at the C-3 position, which is thermodynamically more stable [2,3,4].

Indole alkaloids have gained popularity due to their diverse pharmacological activities. Although both plant and marine sources of indole alkaloids are now being extensively studied worldwide, the present review emphasizes only those indole alkaloids that have been derived from plant sources. Indole alkaloids have been identified in several prominent plant families, including Apocynaceae, Rubiaceae, Nyssaceae, and Loganiaceae, among others. Some of the identified indole alkaloid compounds have been highly effective in pre-clinical and clinical studies. Thousands of compounds containing the indole nucleus have been isolated from plant sources. Their pharmacological activities were assessed, with some now being examined in clinical trials and some already approved for therapeutic use in humans. Indole alkaloids are often characterized by their potent biological activities, which are relevant to the field of medicine, including anticancer, antibacterial, antiviral, antimalarial, antifungal, anti-inflammatory, antidepressant, analgesic, hypotensive, anticholinesterase, antiplatelet, antidiarrheal, spasmolytic, antileishmanial, lipid-lowering, antimycobacterial, and antidiabetic activities [2,3,4].

## 3. Pharmacological Activities

### 3.1. Antimicrobial Activity

Scholarisins I, II, III, and scholarisine F were isolated from the leaves of *Alstonia rupestris*, and the antifungal activities of these compounds were tested by the disk diffusion method. The zones of inhibition and the minimum inhibitory concentrations (MICs) were determined against five species of fungi, and these compounds displayed inhibitory activities against two species of fungi (*Gibberella pulicaris* and *Cercospora nicotianae)*, with MIC values of 0.64–0.69 mM, 1.37–1.44 µM, 1.80–1.91 µM, and 1.55–1.71 µM, respectively [5].

Kopsihainins D, E, F, and kopsiflorine were isolated from the twigs of *Kopsia hainanensis.* The antibacterial activities of these four compounds were examined against *Staphylococcus aureus* using the disk diffusion method. These compounds exhibited inhibitory activity, forming antibacterial regions with diameters of 11.2, 9.1, 10.3, and 9.7 mm, respectively [6].

Erchinines A and B have shown significant antibacterial and antifungal activities against *Trichophyton rubrum* and *Bacillus subtilis.* Both compounds were isolated from the roots of *Ervatamia chinensis*, and they exhibited potent activity, with the MIC values of 0.78 and 0.78 μg/mL against *Bacillus subtilis* and 12.5 and 6.25 μg/mL against *Trichophyton rubrum*, respectively [7].

**Figure 2:**
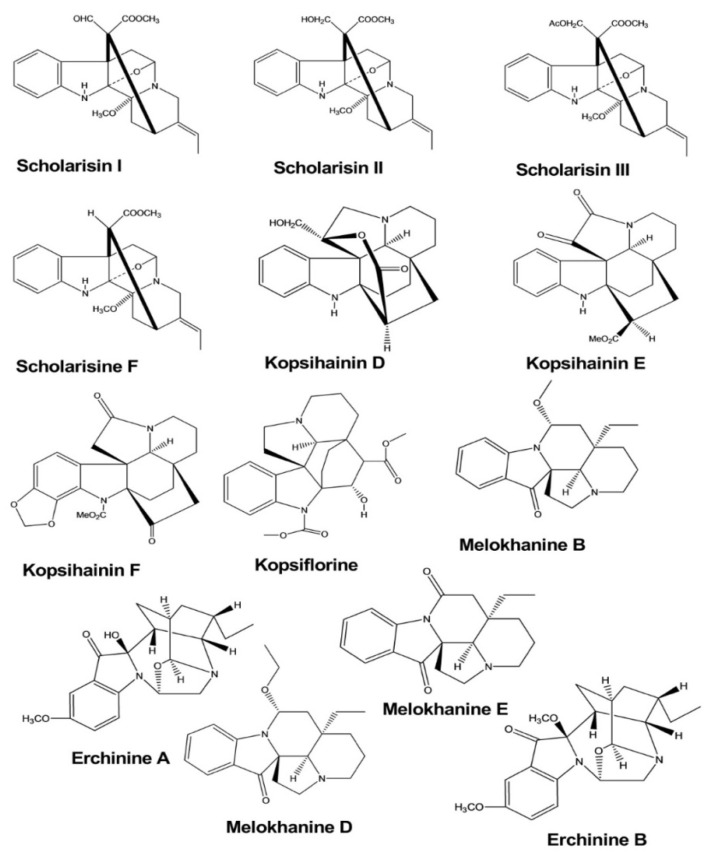
Antimicrobial activity of indole alkaloids.

Melokhanines B, D, E, and F exhibited excellent antibacterial activities against *Pseudomonas aeruginosa*. The MIC values for these compounds were 5, 4, 2, and 2 μM, respectively. Moreover, melokhanine B and E also showed antibacterial activity against *Enterococcus faecalis*, with a MIC value of 5 μM for both compounds (Figure 2) [8].

### 3.2. Antiviral Activity

The compounds 17-nor-excelsinidine and strictamine were extracted from the twigs and leaves of *Alstonia scholaris*. These compounds were shown to significantly inhibit herpes simplex virus (HSV) and adenovirus (ADV), with half-maximal effective concentrations (EC_50_) of 1.09 and 0.36 μg/mL against HSV and 0.94 and 0.28 against ADV, respectively [9].

Trigonoliimine A was extracted from the leaves of *Trigonostemon lii*. This compound was evaluated for anti-HIV-1 activity using a microtiter syncytium formation infectivity assay and was found to exhibit a moderate level of inhibitory activity, with an EC_50_ value of 0.95 µg/mL [10].

**Figure 3:**
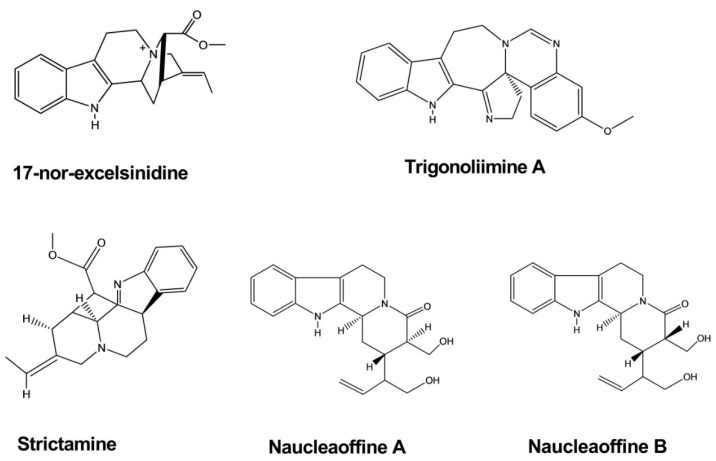
Antiviral activity of indole alkaloids.

Naucleaoffines A and B were isolated from the leaves and stems of *Nauclea officinalis*. Both of these compounds have shown significant effects against HIV-1, with EC_50_ values of 0.06 and 0.23 µM, whereas the positive control demonstrated an EC_50_ value of 0.018 µM (Figure 3) [11].

### 3.3. Antidepressant Activity

Mitragynine is an indole alkaloid isolated from *Mitragyna speciosa Korth*. The antidepressant activity of mitragynine was examined using the forced swim test (FST) and tail suspension test (TST) in a mouse model of depression. Mitragynine significantly decreased the immobility periods of mice in both the FST and TST without noticeable effects on locomotor activity in the open-field test when administered at doses of 10 and 30 mg/kg. The release of corticosterone in mice exposed to the FST and TST was found to be considerably diminished following treatment with mitragynine at doses that provided effective antidepressant effects [12].

Lyaloside and strictosamide exhibited an inhibitory effect against monoamine oxidase (MAO), although this effect was not significant. However, these compounds may represent new leads for the development of analogs with potential antidepressant effects. Lyaloside and stratosamide inhibited MAO-A with half-maximal inhibitory concentrations (IC_50_) values of 50.04 ± 1.09 and 132.5 ± 1.33 μg/mL, respectively, and MAO-B inhibition occurred at IC_50_ values of 306.6 ± 1.40 and 162.8 ± 1.26 μg/mL. Lyaloside and strictosamide were isolated from *Psychotria suterella* and *Psychotria laciniata*, respectively [13].

Harmane, norharmane, and harmine exhibited antidepressant-like activity when administered to mice subjected to the FST. In a dose-dependent manner, these compounds decreased the immobility duration with a 50% effective dose (ED_50_) of 11.5 mg/kg by intraperitoneal (i.p.) administration for harmane, 8.5 mg/kg i.p. for norharmane, and 8 mg/kg i.p. for harmine. These effects do not appear to be mediated by presynaptic monoaminergic mechanisms but are likely caused by an inverse-agonistic mechanism that involves the benzodiazepine receptors [14].

**Figure 4:**
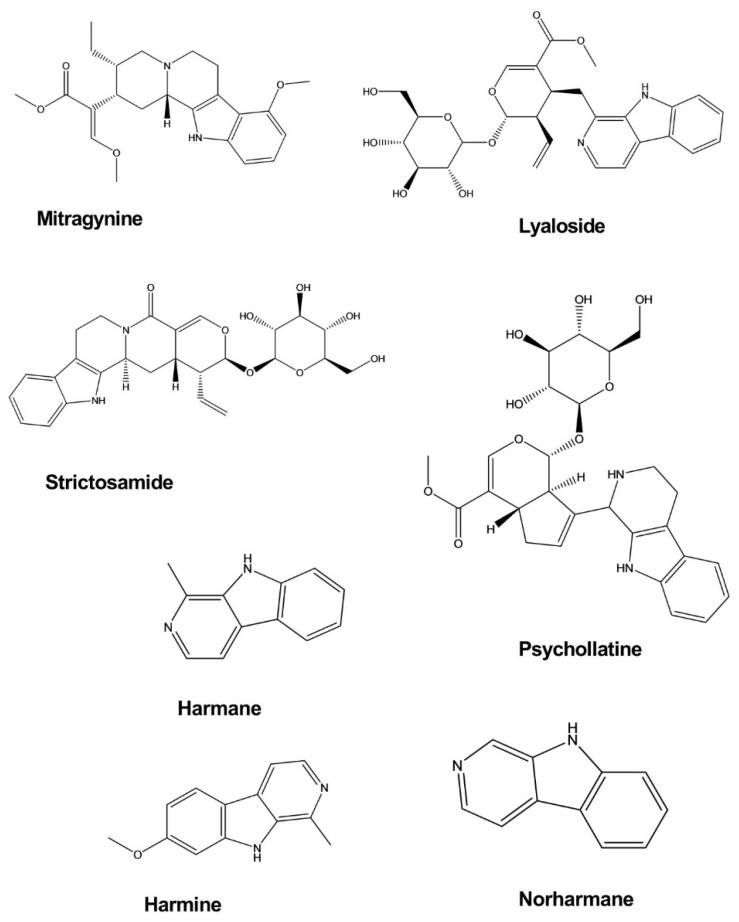
Antidepressant activity of indole alkaloids.

Psychollatine was isolated from the plant *Psychotria umbellate*. Psychollatine increased the number of crossings, rearings, and head-dips of treated mice during the hole-board test at the doses of 7.5 and 15 mg/kg. In the light/dark test, psychollatine increased the time spent in the light area and the latency to the first entry into the dark compartment when administered at a dose of 7.5 mg/kg. In the FST, psychollatine significantly diminished the duration of immobility in mice at doses of 3 and 7.5 mg/kg (Figure 4) [15].

### 3.4. Anticancer Activity

Tabersonine was isolated from the leaves and twigs of *Melodinus fusiformis*. This compound exhibited significant anticancer activity against five human tumor cell lines, with IC_50_ values of 4.6, 5.6, 14.8, 9.9, and 12.1 against SW480, SMMC-7721, HL-60, MCF-7, and A-549 cells, respectively [16].

Brucine displayed significant cytotoxic activity against the human hepatoma cell line HepG2, with IC_50_ values of 0.65, 0.32, and 0.10 mM at a different time interval after treatment. Brucine was isolated from the seeds of *Strychnos nux-vomica* L. Strychnine and isostrychnine, which were extracted from the same plant, also showed cytotoxic activity [17].

Naucleaorals A and B were isolated from the roots of *Nauclea orientalis*. Both compounds showed cytotoxic activity against the KB (human epidermoid carcinoma) and HeLa (human cervical carcinoma) cell lines. However, Naucleaoral A showed substantial cytotoxicity, with an IC_50_ value of 4.0 μg/mL against HeLa cells, whereas Naucleaoral B showed only very moderate cytotoxicity, with IC_50_ values of 7.8 and 9.5 μg/mL against the two cell lines [18].

Vallesiachotamine and iso-vallesiachotamine were isolated from the fruits of *Anthocephalus cadamba (Roxb) Miq*. Both compounds showed potent anticancer activity, with IC_50_ values of 4.24 and 3.79 μM, respectively, against the human lung cancer cell line H1299 after 72 h of incubation [19].

Ervachinines A, C, and D exhibited significant inhibitory effects against five cancer cell lines, including HL-60 human myeloid leukemia cells, SMMC-7721 hepatocellular carcinoma cells, A-549 lung cancer cells, MCF-7 breast cancer cells, and SW480 colon cancer cells. Except for ervachinine D against MCF-7 cells, all three compounds displayed IC_50_ values in the range of 0.84–4.63 μM against all five cancer cell lines. These compounds were extracted from the *Ervatamia chinensis* whole plant [20].

**Figure 5:**
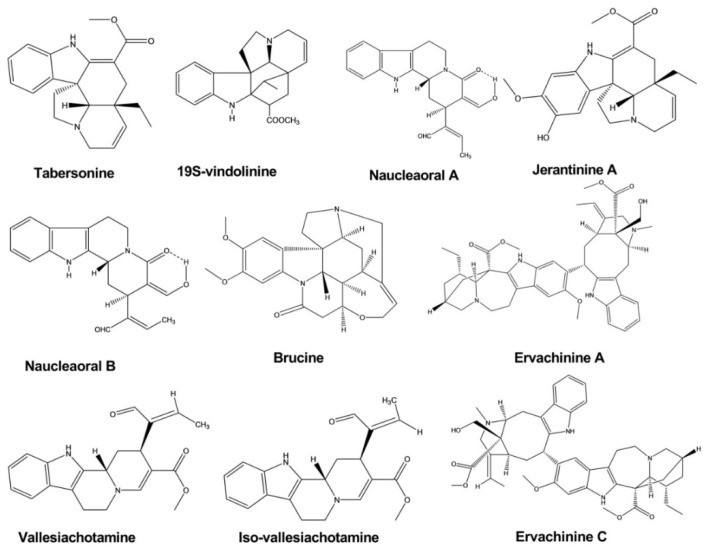
Anticancer activity of indole alkaloids.

Jerantinines A and B were extracted from the plant *Tabernaemontana corymbosa*. These compounds exhibited inhibitory effects against three cancer cell lines, as determined by the 3-(4,5-dimethylthiazol-2-yl)-2,5-diphenyltetrazolium bromide (MTT) assay. The half-maximal growth inhibitory concentration (GI50) was calculated, which showed that jerantinine A displayed an inhibitory effect against breast, colon, and lung carcinoma cell lines, with the GI50 values less than 4.00 μM. Jerantinine B showed significant activity against all cell lines except MCF-7 cells, with GI50 values less than 1.00 μM. Jerantinine A also blocked the ability of cancer cells to form colonies (Figure 5) [21].

### 3.5. Anti-Inflammatory Activity

Melaxillines A and B, two alkaloids containing an indole nucleus, were isolated from the roots of *Melodinus axillaris*. Both compounds showed potent anti-inflammatory activity, as assessed using an in vitro assay to measure the inhibition of β-glucuronidase secretion induced by platelet-activating factor (PAF) in rat polymorphonuclear leukocytes (PMNs). The IC_50_ values of these two compounds were 1.51 and 2.62 μM, respectively [22].

Perakine N_4_-oxide, raucaffrinoline N_4_-oxide, and vinorine N_4_-oxide were isolated from *Alstonia yunnanensis*. An in vitro anti-inflammatory activity assay revealed selective cyclooxidase 2 (COX-2) inhibition by these three compounds, with inhibitory values of 94.77%, 88.09%, and 94.05%, respectively; however, none of these compounds displayed any significant COX-1 inhibition (<45%) [23].

Scholarisins I and VI, two monoterpenoid indole alkaloids, were isolated from *Alstonia rupestris*. Both compounds displayed the selective inhibition of COX-2, with inhibitory values of 96.4% and 95.5%, respectively, with no significant inhibitory effects against COX-1 [5].

**Figure 6:**
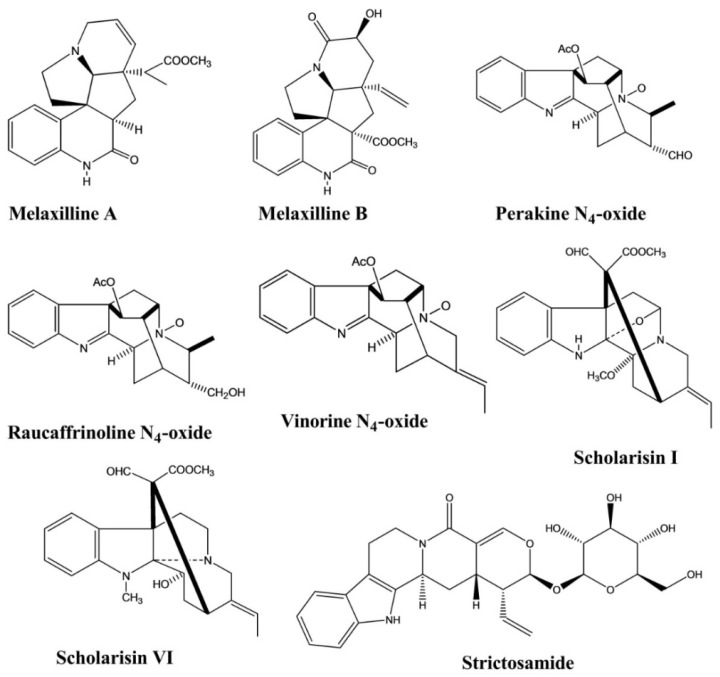
Anti-inflammatory activity of indole alkaloids.

Strictosamide showed anti-inflammatory activity against a mouse model of ear edema induced by 12-O-tetradecanoylphorbol-13-acetate (TPA) at doses of 20 and 40 mg/kg. Strictosamide administration significantly diminished the ear swelling rates from 143.9 ± 8.8 to 108.4 ± 11.7 and 103.5 ± 16.0, representing 24.7% and 28.1% inhibition against inflammation, respectively. Strictosamide substantially blocked peritoneal capillary permeability induced by acetic acid in mice, with inhibitory rates of 23.3% and 33.4% at doses of 20 and 40 mg/kg, respectively. In another test, strictosamide significantly reduced leukocyte counts induced by carboxymethylcellulose sodium (CMC–Na) at doses of 10, 20, and 40 mg/kg, resulting in reductions of 46.0%, 49.1%, and 58.7%, respectively (Figure 6) [24].

### 3.6. Analgesic Activity

Brucine and brucine N-oxide were extracted from the seeds of *Strychnos nux-vomica*. Three different tests, including the hot plate test, writhing test, and formalin test, were conducted to determine whether these compounds exerted analgesic effects. In the formalin test, brucine showed potent inhibitory effects against both the early- and late-phase pain stimuli at doses ranging from 7.5 to 30 mg/kg. However, brucine N-oxide exhibited a significant inhibitory effect only against the late phase. In the writhing test, brucine (15 and 30 mg/kg) and brucine N-oxide (50 and 200 mg/kg) showed significant inhibition of the writhing response to the i.p. administration of acetic acid in mice. In the hot plate test, the ED_50_ value of brucine N-oxide was five and six times greater than that of brucine 30 and 60 min after drug administration, respectively, which indicated that brucine prolonged the pain threshold of mice in a dose-dependent manner [25].

Mitragynine and 7-hydroxymitragynine were isolated from the plant *Mitragyna speciosa* [26,27]. The antinociceptive effects of mitragynine were examined using the hot plate test, which revealed a dose-dependent response at doses ranging from 3–35 mg/kg. The latency period increased after the administration of the 15 mg/kg dose. The most significant antinociceptive effect was observed at the 35 mg/kg mitragynine dose, which corresponded with the longest latency time [26]. Similarly, 7-hydroxymitragynine demonstrated antinociceptive activity in a dose-dependent manner (2.5–10 mg/kg) in the tail-flick and hotplate tests. The maximum possible effect (MPE) value of 7-hydroxymitragynine (5 mg/kg, subcutaneous (s.c.)) reached 100% between 15 and 30 min after administration in the tail-flick test. In the hotplate test, the MPE value of 7-hydroxymitragynine (10 mg/kg, s.c.) reached 94% at 15 min after administration [27].

Strictosamide exhibited analgesic activity in the writhing test, with no such effect observed in the hot plate test. The i.p. injection of strictosamide reduced acetic acid-induced writhing in mice in a dose-dependent manner. Strictosamide remarkably lengthened the pain latency of mice at doses of 20 and 40 mg/kg, resulting in latency periods 336.5 s and 345.8 s, respectively. When the writhing activity was counted, a significant reduction in writhing activity was observed for the 40 mg/kg dose of strictosamide, which reduced the count to 9.7 compared with the positive drug. The inhibition observed at doses of 20 and 40 mg/kg were 37.0% and 49.7%, respectively. This compound was isolated from the *Nauclea officinalis* [24].

**Figure 7:**
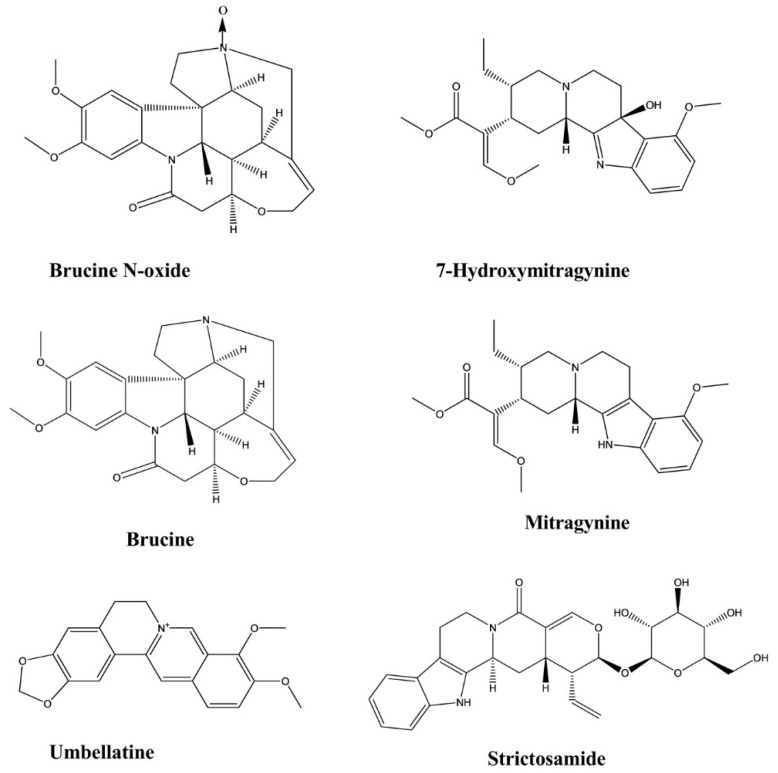
Analgesic activity of indole alkaloids.

Umbellatine was isolated from the leaves of *Psychotria umbellate*. Analgesic activity was investigated by conducting the tail-flick test, hot plate test, formalin test, and capsaicin-induced pain test. In the four test models, umbellatine exhibited good activity against tail-flick test and hot plate test and significant activity against formalin test and capsaicin-induced pain test at the doses of 100–300 mg/kg (Figure 7) [28].

### 3.7. Antidiabetic Activity

Vindoline, vindolidine, vindolicine, and vindolinine represent four new indole-type alkaloids that were extracted from the leaves of *Catharanthus roseus* (L.) G. Don. All four alkaloids induced enhanced glucose uptake activity in β-TC6 and C_2_C1_2_ cells in a dose-dependent manner, and vindolicine triggered an extreme increase in glucose uptake activity. In another test, all of these compounds exhibited protein tyrosine phosphatase 1B (PTP-1B) inhibition activity, although only vindolicine showed significant inhibitory activity. The IC_50_ values of vindoline, vindolidine, vindolicine, and vindolinine against PTP-1B were 36.5, 18.2, 11.6, and 14.1 μM, respectively [29].

Vindogentianine was isolated from *Catharanthus roseus leaf extracts* and resulted in significant hypoglycemic activity in β-TC6 pancreatic and C_2_C1_2_ muscle cells at treatment concentrations of 25.0, 50.0, and 100.0 μg/mL by inducing increased glucose uptake activity. The compound also exhibited a significant antihyperglycemic effect in the PTP-1B inhibition test, with an IC_50_ value of 15.28 ± 2.59 μg/mL [30].

**Figure 8:**
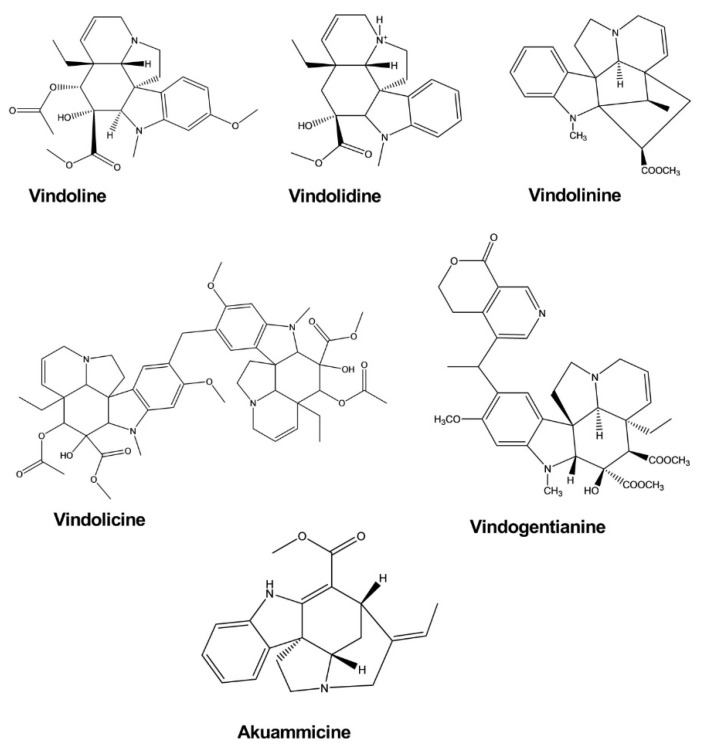
Antidiabetic activity of indole alkaloids.

Akuammicine was isolated from the plant *Picralima nitida* and significantly enhanced glucose uptake activity in fully differentiated 3T3-L1 adipocytes after 24 h incubation (Figure 8) [31].

### 3.8. Antimalarial Activity

Ellipticine and olivacine, two indole alkaloids, exhibited potential in vitro antimalarial activity against *Plasmodium falciparum* and in vivo activity in *Plasmodium berghei*-infected mice. These two compounds were isolated from *Aspidosperma vargasii* and *Aspidosperma olivaceum.* Olivacine significantly inhibited *P. falciparum* growth, with an IC_50_ value of 1.2 μM against the K1 *P. falciparum strain*. Ellipticine displayed significant antimalarial effects, with IC_50_ values of 0.81 and 0.35 μM against the *P. falciparum* strains K1 and 3D7, respectively. Both compounds displayed high selectivity indices against *P. falciparum* 3D7 (>1.2 × 10^3^ and >3.4 × 10^2^, respectively). The in vivo antimalarial activity of these compounds was evaluated in *P. berghei-*infected mice in a four-day suppressive test. Ellipticine showed a significant effect at an oral dose of 50 mg/kg/day (100% inhibition versus controls on days five and seven). The mean survival time (MST) of the animals was greater than 40 days at the same oral dose. Olivacine showed higher activity at the dose equal to 50 mg/kg/day in this test. The range of inhibition was 90%–97% at day five and day seven after the oral and subcutaneous administration of the drug, with an MST of 23–27 days [32].

Flinderoles A, B, and C showed selective antimalarial activity against the Dd2 strain of *P. falciparum*. These compounds exhibited parasite growth inhibition with IC_50_ values of 1.42, 0.15, and 0.34 μM, respectively. These compounds were isolated from *Flindersia acuminate* and *F. amboinensis* [33].

**Figure 9:**
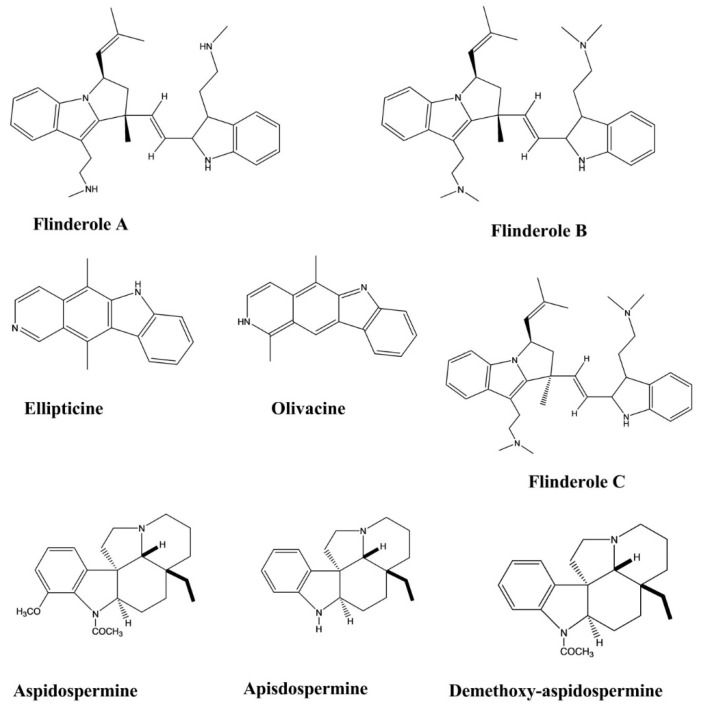
Antimalarial activity of indole alkaloids.

Apisdospermine, aspidospermine, demethoxy-aspidospermine, vallesine, and palosine were tested for their antimalarial activities against a chloroquine-resistant strain of *P. falciparum*. These compounds displayed better activity after incubation for 72 h with IC_50_ values of 3.8, 4.1, 5.6, 6.2, and 12.7 μM, respectively (Figure 9) [34].

**Figure 10:**
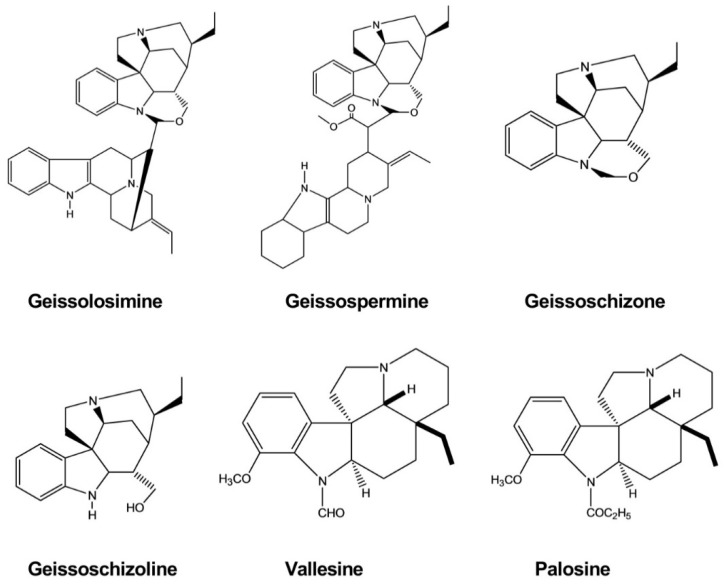
Antimalarial activity of indole alkaloids.

Geissolosimine, geissospermine, geissoschizoline, and geissoschizone were evaluated for in vitro antimalarial activity against the chloroquine-sensitive strain of *P. falciparum* (D10), revealing IC_50_ values of 0.55, 3.17, 4.16, and 3.19 μg/mL, respectively. Among these compounds, geissolosimine exhibited the greatest antiplasmodial activity, suggesting that it may represent a promising lead for antimalarial drug discovery. All of these compounds were isolated from the stem bark of *Geissospermum vellosii* (Figure 10) [35].

### 3.9. Hypotensive Activity

Naucline, angustine, angustidine, nauclefine, and naucletine were isolated from the bark of *the Nauclea officinalis*. These compounds displayed hypotensive effects against phenylephrine (PE)-induced contractions of rat aortic rings associated with intact endothelium. Naucline showed moderate vasorelaxant activity, resulting in 90% relaxation at 1 × 10^−5^ M.The remaining compounds showed significant vasorelaxant activity, resulting in greater than 90% relaxation at 1 × 10^−5^ M in an isolated rat aorta [36].

Alstilobanines A, B, and C and undulifoline displayed slow relaxation activity against PE-induced contractions in thoracic rat aortic rings with intact endothelium. The percentages of relaxation induced by these compounds were 44.3%, 21.2%, 28%, and 33.3%, respectively, with alstilobanine A showing more potent relaxation activity than the other compounds. The first three compounds were isolated from *Alstonia angustiloba*, whereas undulifoline was extracted from *Alstonia undulifolia* [37].

Taberniacins A and B, two new indole alkaloids, were isolated from *Tabernaemontana divaricata* and displayed hypotensive activity by inducing vasorelaxation to counteract the PE-induced contraction in an isolated rat aorta. Both alkaloids exhibited moderate levels of vasorelaxant activity in an isolated rat aorta. The IC_50_ values of taberniacins A and B were 2.86 μM and 580 nM, respectively, and vasorelaxation activity increased in a concentration-dependent manner [38].

**Figure 11:**
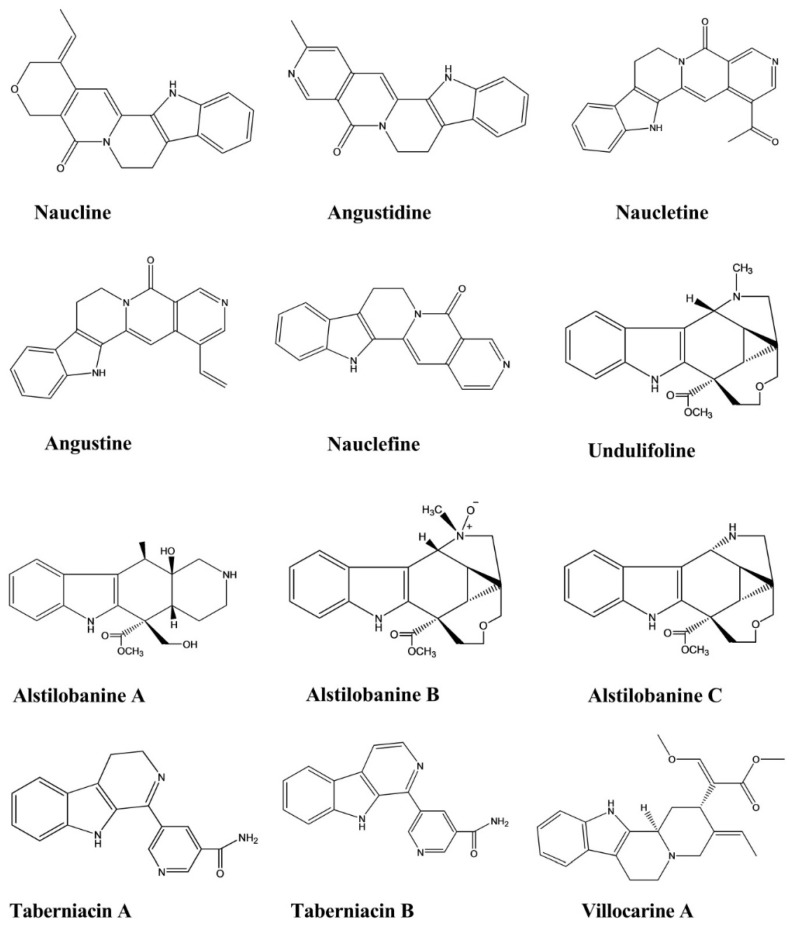
Hypotensive activity of indole alkaloids.

Villocarine A demonstrated vasorelaxation activity in a rat aortic ring, with concentration-dependent inhibitory effects against vasocontraction in aorta depolarized by a high potassium concentration and against PE-mediated contraction in the presence of nicardipine. Villocarine A exhibited excellent activity during the initial stage, within 10–30 min after injection, with potent vasorelaxant effects observed at 30 μM against PE-mediated contraction in a rat aorta. Villocarine A exhibited a moderate level of inhibition at 30 μM against PE and 1 μM-induced contraction of the aortic rings in the presence of nicardipine (1 μM) in a Ca^2+^-free medium. Villocarine showed slightly less vascular relaxation activity in aortic tissues with no endothelium. Villocarine A was isolated from *Uncaria villosa* (Figure 11) [39].

### 3.10. Anticholinesterase Activity

Macusine B, vinorine, isoreserpiline, and rescinnamine are four indole alkaloids isolated from the bark of *Rauvolfia reflexa*. These compounds showed inhibitory activities against the cholinesterase enzyme, with the IC_50_ values of 48.39, 35.06, 24.89, and 11.01 μM, respectively. Rescinnamine displayed the most significant anticholinesterase activity among these four compounds [40].

Voacangine hydroxyindolenine and rupicoline were identified in the chloroform extract of *Tabernaemontana australis stalks*. These compounds were investigated for their anticholinesterase activity and displayed activities at concentrations similar to those of the standard compounds physostigmine and galanthamine [41].

Coronaridine and voacangine were isolated from the stems of *Ervatamia hainanensis*. These compounds displayed significant inhibitory activities against the cholinesterase enzyme, with IC_50_ values of 8.6 and 4.4 μM, respectively, in in vitro experiments [42].

**Figure 12:**
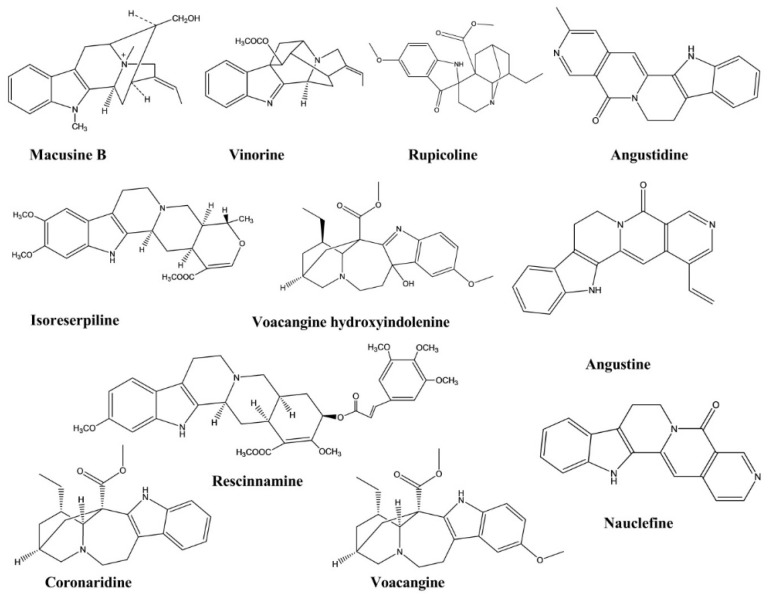
Anticholinesterase activity of Indole alkaloids.

Angustidine, nauclefine, and angustine, three indole alkaloids, were extracted from the plant *Nauclea officinalis*. Their cholinesterase inhibitory activities were evaluated, and all three compounds demonstrated anticholinesterase activity, with IC_50_ values of 1.03, 7.70, and 4.98 μM, respectively, against the butyrylcholinesterase enzyme (Figure 12) [43].

### 3.11. Antiplatelet Activity

**Figure 13:**
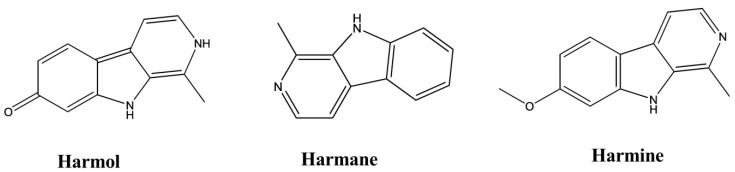
Antiplatelet activity of indole alkaloids.

Harmane, harmine, and harmol were isolated from the plant *Perganum harmala L*. The antiplatelet activities of these three compounds were examined using the selective inhibition of collagen-mediated platelet activation. The IC_50_ values of these three compounds were 113.7 ± 8.4, 132.9 ± 16.6, and 200 ± 4.6 μM, respectively. The underlying mechanism of this effect was also proposed in that study. These compounds had no inhibitory effects on arachidonic acid- or thrombin-induced platelet aggregation at concentrations of 200 μM (Figure 13) [44].

### 3.12. Antidiarrheal Activity

Kurryam and koenimbine were isolated from the seeds of *Murraya koenigii Spreng*. These compounds showed significant antidiarrheal activity in a castor oil-induced diarrhea rat model. The results showed that the mean defecation of rats treated with koenimbine at doses of 10, 30, and 50 mg/kg were 2.51 ± 0.58, 1.94 ± 0.81, and 1.29 ± 0.21, respectively, whereas rats treated with the same dose of kurryam had mean defecation of 2.35 ± 0.35, 1.88 ± 0.28, and 1.21 ± 0.25, respectively [45].

**Figure 14:**
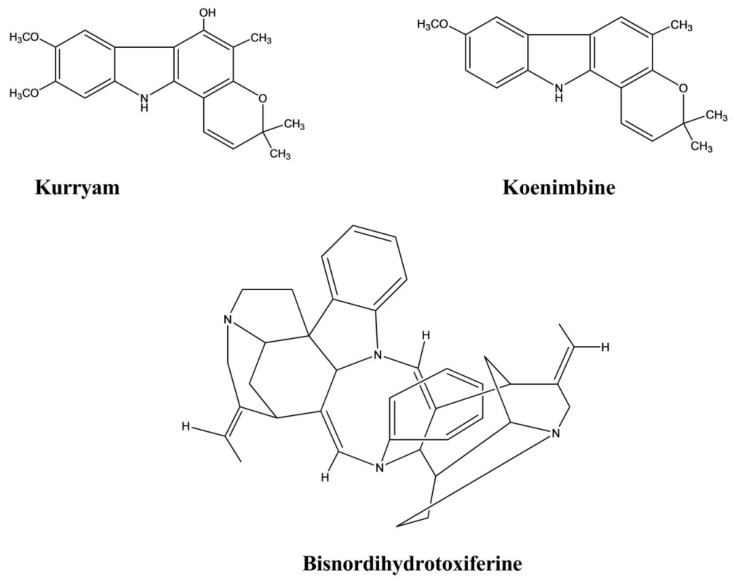
Antidiarrheal activity of indole alkaloids.

Bisnordihydrotoxiferine was extracted from the root bark of *Strychnos trinervis (Vell.) Mart*. The antidiarrheal effect was evaluated in castor oil-, magnesium sulfate-, and arachidonic acid-induced diarrhea models, and activity was calculated as the percent inhibition. This compound inhibited castor oil-induced diarrhea with an inhibition percentage ranging from 70.0% to 100.0% at various doses. Bisnordihydrotoxiferine exhibited 92.5% inhibition at a dose of 25 mg/kg against magnesium-induced diarrhea and 94.6% inhibition at the 12.5 mg/kg dose against arachidonic acid-induced diarrhea (Figure 14) [46].

### 3.13. Spasmolytic Activity

Trinervine is a tertiary indole alkaloid extracted from the root bark of *Strychnos trinervis*. The spasmolytic activity was evaluated for this compound using four different methods, including arachidonic acid (AA)- and 5-hydroxytryptamine (5-HT)-induced contractions of the rat fundic strip and the histamine- and carbachol-mediated contractions of the guinea-pig ileum. The values (mean ± standard error) of the antagonistic potency of trinervine against these four models were 3.96 ± 0.16 (AA), 3.54 ± 0.15 (5-HT), 4.25 ± 0.16 (Histamine), 4.04 ± 0.12 (Carbachol). Trinervine yielded a non-competitive, antagonistic, spasmolytic activity on isolated gastrointestinal smooth muscles [47].

**Figure 15:**
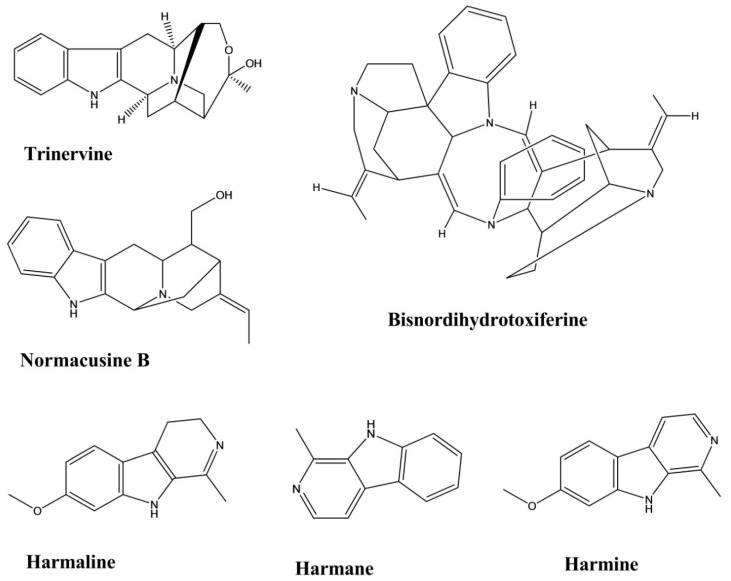
Spasmolytic activity of indole alkaloids.

Bisnordihydrotoxiferine was extracted from the root of *Strychnos diuaricuns*. Acetylcholine- and oxytocin-induced contractions of the rat uterus and acetylcholine- and histamine-induced contractions of the guinea-pig ileum were used to evaluate the contraction inhibition mediated by bisnordihydrotoxiferine. The percentages of inhibition against acetylcholine- and oxytocin-induced contractions in the rat uterus ranged from 21.1% to 77.4% and from 36.6% to 85.0%, respectively, at three different doses. The percentages of inhibition against contractions caused by acetylcholine and histamine in the guinea-pig ileum ranged from 25.0% to 57.2% and 51.5% to 91.1%, respectively, at three different doses (Figure 15) [48].

Normacusine B is a tertiary indole alkaloid that was isolated from the root bark of *Strychnos atlantica*. Normacusine B reduced PE- and serotonin-induced contractions in rat aortic rings, with a molar antagonist potency (*p*A2) value of 7.05 ± 0.11, and non-competitively inhibits 5-HT-induced contractions with a *p*A2 value of 7.02 ± 0.08 [49].

Harmine, harman, and harmaline were isolated from the seeds of *Peganum harmala L*. The relaxation potency of these compounds against histamine, carbachol, and KCl-induced contractions revealed EC50 values for harmane, harmine, and harmaline of 28 ± 3, 16 ± 4, and 20 ± 3 μM against carbachol; 21 ± 2, 10 ± 2, and 143 ± 20 μM against histamine, 51 ± 5, 37 ± 3, and 131 ± 30 μM against KCl, respectively. Harmine displayed the most potency among the three indole alkaloids (Figure 15) [50].

### 3.14. Antileishmanial Activity

**Figure 16:**
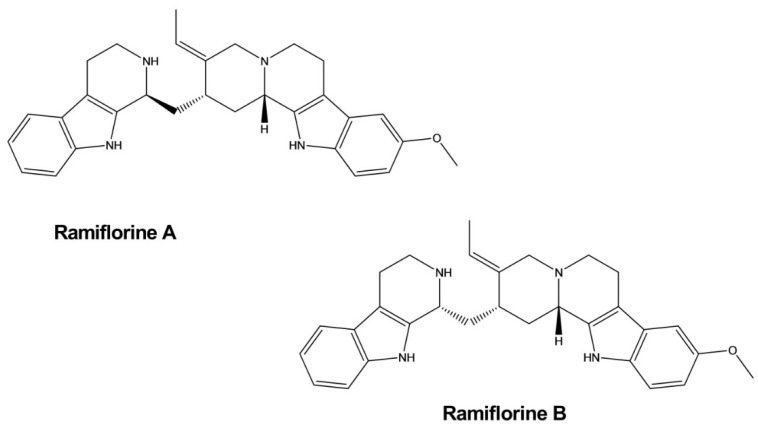
Antileishmanial activity of indole alkaloids.

Ramiflorines A and B were isolated from *Aspidosperma ramiflorum*. These two indole alkaloids exhibited significant effects against *Leishmania amazonensis*, a parasite that causes a disease known as leishmaniasis. Ramiflorines A and B displayed inhibitory activity against the parasite, with LD_50_ values of 16.37 ± 1.6 and 4.97 ± 0.9 mg/mL, respectively, which was 3–10-fold higher than the impacts of the whole-plant alkaloidal extract. Their modes of action were not determined but might be similar to the effects exerted by other corynanthe dimeric indole alkaloids (Figure 16) [51].

### 3.15. Lipid-Lowering Activity

Ellipticine and 9-methoxyellipticine were isolated from *Ochrosia borbonica*. A triglyceride assay in the 3T3-L1 adipocyte model was used to evaluate the lipid-lowering activity of these two compounds. The results indicated that both compounds significantly reduced the formation of lipid droplet by 80% at 10 µmol × L^−1^ and exerted a dose-dependent reduction in lipid formation at a concentration range of 0.01–10 µmol × L^−1^. The EC_50_ values of ellipticine and 9-methoxyellipticine were 0.41 and 0.92 µmol × L^−1^, respectively. This activity might be due to the retardation of adipogenesis and lipogenesis through intercalation into supercoiled DNA [52].

**Figure 17:**
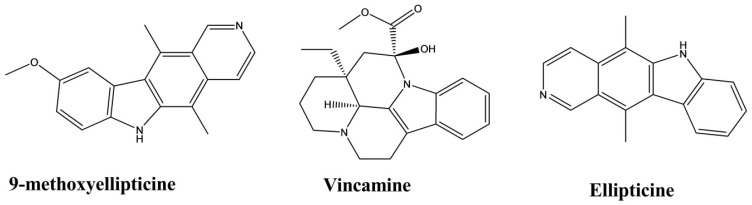
Lipid-lowering activity of indole alkaloids.

Vincamine was isolated from the plant *Vinca minor* [53]. At doses of 20 and 30 mg/kg, vincamine exhibited significant reductions in the levels of serum triglycerides (TG), total cholesterol (TC), low-density lipoprotein cholesterol (LDL-C), and very-low-density lipoprotein cholesterol (VLDL-C), and a comparative increase in high-density lipoprotein cholesterol (HDL-C), resulting in the overall improvement of the lipid profile of rats (Figure 17) [54].

### 3.16. Antimycobacterial Activity

Coronaridine is an iboga-type indole alkaloid isolated from the root of *Tabernaemontana ternifolia* that displayed weak inhibitory activity against *Mycobacterium tuberculosis*. Coronaridine exhibited 90% inhibition against the bacterium, with a MIC of 82.64 µg/mL [55].

Globospiramine, a new spirobisindole alkaloid that displayed significant antimycobacterial activity against *Mycobacterium tuberculosis*, was isolated from *Voacanga globosa*. This compound exhibited potent inhibitory activity against the bacterium, with a MIC of 4.00 µg/mL in a microplate Alamar blue assay and a MIC of 5.20 µg/mL in a low-oxygen recovery assay [56].

**Figure 18:**
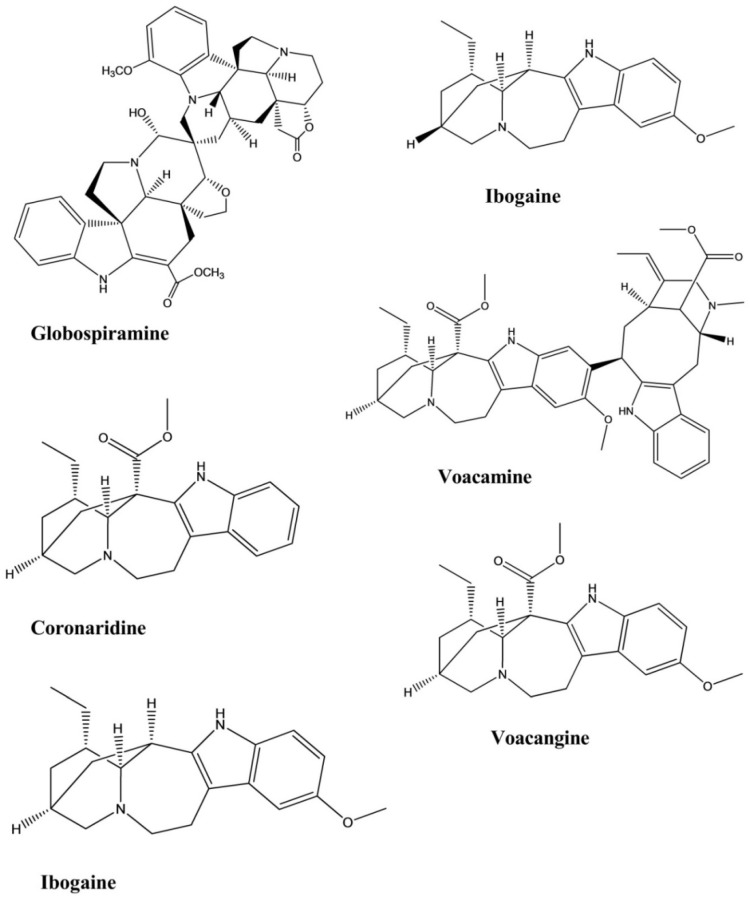
Antimycobacterial activity of indole alkaloids.

Ibogaine and voacangine were isolated from the plant *Tabernaemontana citrifolia* and were evaluated for their antimycobacterial activity against *Mycobacterium tuberculosis*. Both of these compounds displayed inhibitory effects against the bacterium, with MICs of 50.00 µg/mL for each (Figure 18) [57].

**Figure 19:**
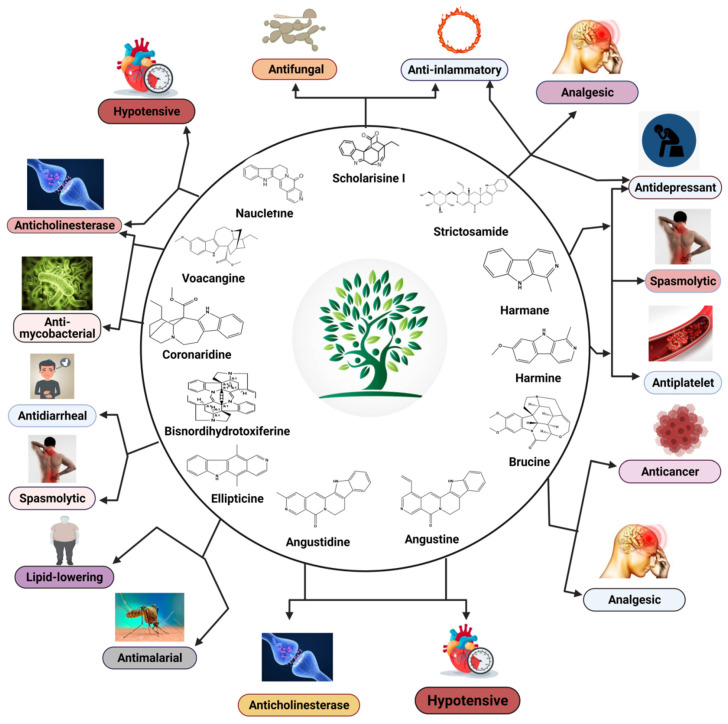
An overview of the pharmacological activities of major indole alkaloids.

Voacamine is a bis-indole-type alkaloid isolated from the root of *Tabernaemontana arborea*. The compound showed inhibitory activity against the causative agent of tuberculosis, *Mycobacterium tuberculosis*. The results showed that the MIC and the IC_50_ values were 15.60 and 16.30 µg/mL, respectively (Table 1, Figure 19) [58].

## 4. Pharmacokinetic Profile

A pharmacokinetics (pk) study of a compound describes four basic phenomena that occur when a foreign substance (xenobiotic) enters the body: absorption, distribution, metabolism or biotransformation, and excretion. The kinetics of these four phenomena help researchers understand, analyze, and predict the biological activities of xenobiotics [59].

Absorption describes the process through which a drug enters the body’s circulation at the site of administration across biological membranes. The rate and extent of absorption depend on the route and the site of administration, and the chemical properties of the drug. Absorption directly affects the bioavailability of substances in the blood and can occur through various mechanisms, including passive and facilitated diffusion, active transport, endocytosis, and exocytosis. Distribution is the next step, during which substances from the bloodstream enter into tissues. Distribution is affected by cardiac output, local blood flow, capillary permeability, tissue volume, regional pH, the degree of plasma binding, and the substances’ relative lipophilicity. Metabolism is a fundamental process through which a substance is chemically altered within the body to facilitate excretion, which typically occurs in the liver. Metabolism involves Phase I and Phase II reactions. Although the primary goal of this process is to facilitate excretion, metabolism can convert the active form of a substance into an inactive form or activate a previously inactive form, as in the case of prodrugs. After metabolism, substances leave the body through a process known as excretion, which can occur through many methods, including in urine, feces, sweat, milk, and expired air.

Some of the compounds reviewed in this article were subjected to pk studies (Table 2).

Brucine is among the major indole alkaloids with various potent pharmacological activities. The pk properties of brucine were investigated after intravenous and oral administration to rats, which resulted in some significant outcomes. The determination of the apparent partition coefficient, plasma protein binding, and other pk properties were evaluated. Brucine showed no sign of degradation in gastrointestinal pH conditions, and the total concentration of the drug in both the water and oil phase did not change after 2 h of incubation at pH 1–7.8, indicating relative stability under acidic conditions. The pH has a substantial effect on the apparent partition coefficient, particularly in the range of pH 6–7.8. Brucine showed a partition coefficient below 0.1 due to complete protonation at pH 1–5. The apparent partition coefficient of brucine under different pH conditions indicated that the most likely absorption site was the intestine because brucine became ionized under stomach-like pH conditions. The protein binding assay revealed that the majority of brucine was primarily bound to the rat plasma protein, with the unbound fraction representing 34.4% ± 3.0%, 35.1% ± 2.9%, and 40.4% ± 2.7% of the total at treatment concentrations of 500, 1250, and 2500 μg/L, respectively. After administering single intravenous doses of 2.5, 5, and 10 mg/kg, the ratio of the mean area under the curve (AUC) values was 1:1.9:5.3 when the dose increased at a ratio of 1:2:4. The total body clearance drastically decreased at the 10 mg/kg dose, whereas the terminal elimination half-life (T1/2) increased in a dose-dependent fashion. Brucine was rapidly absorbed (Tmax = 0.3–0.5 h) and achieved a mean maximum serum concentration (Cmax) between 929.22 and 1451.58 μg/L after oral administration. The absolute oral bioavailability was 40.31%–47.15%. The increase in AUC was proportional to the increase in dose. Both the oral and intravenous administration routes for brucine showed non-linear pk properties overall [60].

A single dose of 40 mg mitragynine (kg/body wt.) was given to six rats, and a non-compartmental pk analysis was performed. Mitragynine was rapidly absorbed after oral administration, reaching a Cmax of 0.63 ± 0.18 µg/mL at 1.83 ± 1.25 h (Tmax), with an absorption rate constant (ka) of 1.43 ± 0.90 h−1. Mitragynine showed a high volume of distribution (Vd/F, 89.50 ± 30.30 L/kg) due to distribution into highly perfused and lipid-containing tissues, specifically the brain, which is the site of action for mitragynine. Mitragynine displayed a slow elimination, with an elimination rate constant (λz) of 0.07 ± 0.01 h^−1^ and a clearance rate (Cl/F) of 1.60 ± 0.58 L/h. The half-lives of absorption (t1/2 ab) and elimination (t1/2 λz) were 0.48 ± 0.36 and 9.43 ± 1.74 h, respectively. The mean residence time (MRT0→∞) was 14.00 ± 2.84 h [61]. Another study showed that mitragynine experienced 26% degradation in simulated gastric fluid, indicating that mitragynine was unstable in gastric fluid, whereas stability was demonstrated in simulated intestinal fluid. Another mitragynine-like compound, 7-hydroxymitragynine, was degraded by up to 27% in simulated gastric juice, which might be due to conversion to mitragynine (23%), whereas only 6% degradation was observed in simulated intestinal fluid. Both of these compounds exhibited greater than 90% protein binding, as determined by the equilibrium analysis. In addition, 7-hydroxymitragynine was shown to have moderate intestinal and blood–brain barrier (BBB) permeability with a significant conversion to mitragynine. It showed an intermediate level of intestinal absorption. It was rapidly metabolized by Phase I metabolic enzymes, with a short T1/2 of 24 min. The high clearance of 43.2 ± 3.5 mL/min/kg in the presence of human liver microsomes results in low bioavailability and limited distribution to tissues. Mitragynine and 7-hydroxymitragynine exhibited significant P-gp (P-glycoprotein) inhibition, similar to verapamil, indicating the possibility of drug-drug interactions when coadministered with drugs that are P-gp substrates [62].

The pk study of vindoline was characterized by the use of a non-compartmental method. Doses of 15 mg/kg (oral), 3 mg/kg (IV), and 6 mg/kg (IV) of vindoline were administered to rats. The area under the plasma concentration-time curves, AUC (0-t) and AUC (0-∞,) were 606.3 and 609.1 ng/mL h, respectively for the oral dose, 2245.7 and 3776.2 ng/mL h for the low-dose IV administration, and 2258.0 and 3788.4 ng/mL h for the high-dose IV administration. For the oral dose, the T1/2, the plasma clearance (CL), and the apparent volume of distribution (V) were 0.5 h, 26.6 L/h/kg, and 21.6 L/kg, respectively. For the IV doses, the T1/2, CL, and V values were 1.0 h, 1.4 L/h/kg, and 2.0 L/kg, respectively, for the 3 mg/kg concentration and 1.4 h, 1.6 L/h/kg, and 3.3 L/kg for the 6 mg/kg concentration, indicating a dose-dependent increase in these parameters. The Cmax and Tmax were calculated, resulting in values of 606.6 ng/mL and 0.3 h, respectively, for the oral dose. For the IV vindoline administration, a dose-dependent increase in Cmax was observed, with values of 1595.9 and 2913.9 ng/mL, respectively, observed for the 3 and 6 mg/kg concentrations. The bioavailability of vindoline after oral administration was 5.4% [63].

Harmane was immediately absorbed into the blood circulation, with a high Cmax of 1059.56 ± 91.06 ng/mL and a short Tmax of 0.23 ± 0.06 h after the administration of a single oral dose at 30.0 mg/kg body weight in rats. The plasma concentration-time curve for harmane displayed a rapid decrease, with an elimination half-life (T1/2e) of 2.26 ± 0.53 h, and the levels fell below the detection limits within 8 h after administration. The oral bioavailability of harmane was 19.41%. The absorption rate constant (Ka), distribution rate constant (Kd), and elimination rate constant (Ke) were 3.64, 1.51, and 0.32 per hour. Other parameters were also evaluated in that study. After an intravenous bolus administration of harmane at a dose of 1.0 mg/kg, the harmane plasma concentration versus time curve yielded a sharp decline in the concentration, followed by a fast phase of decrease, with a T1/2e of 4.71 ± 1.46 h, until the levels fell below the detection limits. The volume of distribution (Vd) value for harmane was relatively high. The Cmax value was 583.19 ng/mL at a Tmax of 0.03 h. The Ka, Kd, and Ke values were 4.18, 1.54, and 0.16 per hour. The metabolic process for harmane was also determined, and sulfate conjugation appeared to be the most prominent process [64].

## 5. Conclusions and Future Perspectives

Indole alkaloids of plant origin have demonstrated diverse potential pharmacological activities; therefore, we reviewed various indole compounds and discussed their respective pharmacological importance. Although the therapeutic importance of these compounds has been evaluated, in many cases, an insufficient number of studies have examined their efficacy and safety. The effects demonstrated by various compounds, including strictosamide (antidepressant, analgesic, and anti-inflammatory), mitragynine (analgesic and antidepressant), harmane (antidepressant, spasmolytic, and antiplatelet), brucine (analgesic and anticancer), ellipticine (antimalarial and lipid-lowering), globospiramine (antimycobacterial), vindogentianine (antidiabetic), tabersonine (anticancer), scholarisine I (antifungal and anti-inflammatory), angustine (anticholinesterase and hypotensive), suggest that further in-depth studies should be performed to endorse the clinical effectiveness of these compounds. In addition, the mechanistic activities at the sites of action and toxicity studies should be thoroughly investigated to identify suitable lead compounds for further development. Due to the lack of extensive pharmacological information regarding these compounds obtained from previous studies, new clinical studies that are performed using well-developed methodologies remain necessary to identify new therapeutic agents with increased efficacy and reduced side effects. The pk properties of some compounds have also shown promising results that should be explored further. This review can be useful for researchers who plan to extensively study indole alkaloids in the future.

## Figures and Tables

**Table 1:** Plant source and pharmacological activity of indole compounds.

Compounds	Plant Source	Posology (Route, Dose)	Subject	Method	Identified Effect	References
Scholarisine I, II, III, F	*Alstonia rupestris*	-	Fungi	Disc diffusion method	Antifungal	[5]
Scholarisine I, VI	*Alstonia rupestris*	-	Enzyme	Enzyme inhibition assay	Anti-inflammatory	[5]
Kopsihainin D, E, F	*Kopsia hainanensis*	-	Bacteria	Disc agar diffusion method	Antibacterial	[6]
Kopsiflorine	*Kopsia hainanensis*	-	Bacteria	Disc agar diffusion method	Antibacterial	[6]
Erchinine A, B	*Ervatamia chinensis*	-	Bacteria Fungus	Broth microdilution method	Antibacterial, Antifungal	[7]
Melokhanine B, D, E, F	*Melodinus khasianus*	-	Bacteria	Broth microdilution method	Antibacterial	[8]
Melokhanine B, D, E, F	*Melodinus khasianus*	-	Bacteria	Broth microdilution method	Antibacterial	[8]
Strictamine	*Alstonia scholaris*	-	Virus	-	Antiviral	[9]
17-nor-excelsinidine	*Alstonia scholaris*	-	Virus	-	Antiviral	[9]
Trigonoliimine	*Trigonostemon lii*	-	Virus	Microtiter syncytium formation infectivity assay	Antiviral	[10]
Naucleaoffine A, B	*Nauclea officinalis*		Virus		Antiviral	[11]
Mitragynine	*Mitragyna speciosa*	Intraperitoneal, 10 and 30 mg/kg	Mice	Forced swim test, Tail suspension test	Antidepressant	[12]
Lyaloside	*Psychotria suterella*	-	Rat brain mitochondria	Enzymatic assay	Antidepressant	[13]
Strictosamide	*Psychotria laciniata*	-	Rat brain mitochondria	Enzymatic assay	Antidepressant	[13]
Harmane	*Peganum harmala*	Intraperitoneal, 5–15 mg/kg	Mice	Forced swim test	Antidepressant	[14]
Norharmane	*Peganum harmala*	Intraperitoneal, 2.5–10 mg/kg	Mice	Forced swim test	Antidepressant	[14]
Harmine	*Peganum harmala*	Intraperitoneal, 5–15 mg/kg	Mice	Forced swim test	Antidepressant	[14]
Psychollatine	*Psychotria umbellate*	3, 7.5, and 15 mg/kg	Mice	Hole-board test, Forced swim test	Antidepressant	[15]
Tabersonine	*Melodinus fusiformis*	-	Human tumor cell line	MTT assay	Anticancer	[16]
Brucine	*Strychnos nux-vomica*	-	Human hepatoma cell line	MTT-colorimetric assay	Anticancer	[17]
Naucleaoral A, B	*Nauclea orientalis*	-	Human cancer cell line	MTT-colorimetric assay	Anticancer	[18]
Vallesiachotamine	*Anthocephalus cadamba*	-	Human lung cancer cell line	MTT assay	Anticancer	[19]
Iso-vallesiachotamine	*Anthocephalus cadamba*	-	Human lung cancer cell line	MTT assay	Anticancer	[19]
Ervachinine A, C, D	*Ervatamia chinensis*	-	Human cancer cell line	MTT assay	Anticancer	[20]
Jerantinine A, B	*Tabernaemontana corymbosa*		Human cancer cell line	MTT assay	Anticancer	[21]
Melaxilline A, B	*Melodinus axillaris.*	-	Rat	Platelet-activating factor induced inhibition assay	Anti-inflammatory	[22]
Perakine N_4_-oxide	*Alstonia yunnanensis.*	-	Enzyme	Enzyme inhibition assay	Anti-inflammatory	[23]
Raucaffrinoline N_4_-oxide	*Alstonia yunnanensis.*	-	Enzyme	Enzyme inhibition assay	Anti-inflammatory	[23]
Vinorine N_4_-oxide	*Alstonia yunnanensis.*	-	Enzyme	Enzyme inhibition assay	Anti-inflammatory	[23]
Strictosamide	*Nauclea officinalis*	Intraperitoneal, 20 and 40 mg/kg	Mice	Hot plate test, Writhing test	Analgesic	[24]
Strictosamide	*Psychotria laciniata*	Intravenous, 20 and 40 mg/kg	Mice	Acetic acid and TPA-induced assay	Anti-inflammatory	[24]
Brucine	*Strychnos nux-vomica*	Intraperitoneal, 30, 15, and 7.5 mg/kg	Mice	Formalin test	Analgesic	[25]
Brucine	*Strychnos nux-vomica*	Intraperitoneal, 30, 20, 14.7, and 10.3 mg/kg	Mice	Hot plate test	Analgesic	[25]
Brucine	*Strychnos nux-vomica*	Intraperitoneal, 30, 15, 7.5, and 3.75 mg/kg	Mice	Writhing test	Analgesic	[25]
Brucine N-oxide	*Strychnos nux-vomica*	Intraperitoneal, 200, 100 and 50 mg/kg	Mice	Formalin test	Analgesic	[25]
Brucine N-oxide	*Strychnos nux-vomica*	Intraperitoneal, 200, 140, 98, and 68 mg/kg	Mice	Hot plate test	Analgesic	[25]
Brucine N-oxide	*Strychnos nux-vomica*	Intraperitoneal, 200, 100, 50, and 25 mg/kg	Mice	Writhing test	Analgesic	[25]
Mitragynine	*Mitragyna speciosa*	Intraperitoneal, 3–35 mg/kg	Mice	Hot plate test	Analgesic	[26]
7-hydroxymitragynine	*Mitragyna speciosa*	Subcutaneous, 2.5–10 mg/kg	Mice	Hot plate test, Tail flick test	Analgesic	[27]
Umbellatine	*Psychotria umbellate*	100–300 mg/kg	Mice	Tail-flick test, Hot-plate test, Formalin test and Capsaicin-induced pain test	Analgesic	[28]
Vindoline	*Catharanthus roseus*	-	Enzyme, Myoblast cell	Enzyme inhibition assay, Glucose uptake activity assay	Antidiabetic	[29]
Vindolidine	*Catharanthus roseus*	-	Enzyme, Myoblast cell	Enzyme inhibition assay, Glucose uptake activity assay	Antidiabetic	[29]
Vindolicine	*Catharanthus roseus*	-	Enzyme, Myoblast cell	Enzyme inhibition assay, Glucose uptake activity assay	Antidiabetic	[29]
Vindolinine	*Catharanthus roseus*	-	Enzyme, Myoblast cell	Enzyme inhibition assay, Glucose uptake activity assay	Antidiabetic	[29]
Vindogentianine	*Catharanthus roseus*	-	Enzyme, Myoblast cell	Enzyme inhibition assay, Glucose uptake activity assay	Antidiabetic	[30]
Akuammicine	*Picralima nitida*	-	Myoblast cell	Glucose uptake activity assay	Antidiabetic	[31]
Ellipticine	*Aspidosperma vargasii*	Oral, Subcutaneous, 50, 10 and 1 mg/kg/day	Mice	Suppressive test	Antimalarial	[32]
Olivacine	*Aspidosperma olivaceum.*	Oral, Subcutaneous, 100, 50, 10, and 1 mg/kg/day	Mice	Suppressive test	Antimalarial	[32]
Flinderole A, B, C	*Flindersia acuminate* *F. amboinensis*	-	Malarial Parasite	Micrdilution method	Antimalarial	[33]
Apisdospermin		-	Malarial Parasite	Micrdilution method	Antimalarial	[34]
Aspidospermine	*Aspidosperma pyrifolium*	-	Malarial Parasite	Micrdilution method	Antimalarial	[34]
Demethoxy-aspidospermine	*Aspidosperma pyrifolium*	-	Malarial Parasite	Micrdilution method	Antimalarial	[34]
Vallesine	*Aspidosperma pyrifolium*	-	Malarial Parasite	Micrdilution method	Antimalarial	[34]
Palosine	*Aspidosperma pyrifolium*	-	Malarial Parasite	Micrdilution method	Antimalarial	[34]
Geissolosimine	*Geissospermum vellosii*	-	Malarial Parasite	Parasite lactate dehydrogenase assay	Antimalarial	[35]
Geissospermine	*Geissospermum vellosii*	-	Malarial Parasite	Parasite lactate dehydrogenase assay	Antimalarial	[35]
Geissoschizoline	*Geissospermum vellosii*	-	Malarial Parasite	Parasite lactate dehydrogenase assay	Antimalarial	[35]
Geissoschizone	*Geissospermum vellosii*	-	Malarial Parasite	Parasite lactate dehydrogenase assay	Antimalarial	[35]
Naucline	*Nauclea* *officinalis*	Injection, 1 × 10^−5^ M	Rat	Phenylephrine-induced vasodilation assay	Hypotensive	[36]
Angustine	*Nauclea* *officinalis*	Injection, 1 × 10^−5^ M	Rat	Phenylephrine-induced vasodilation assay	Hypotensive	[36]
Angustidine	*Nauclea* *officinalis*	Injection, 1 × 10^−5^ M	Rat	Phenylephrine-induced vasodilation assay	Hypotensive	[36]
Nauclefine	*Nauclea* *officinalis*	Injection, 1 × 10^−5^ M	Rat	Phenylephrine-induced vasodilation assay	Hypotensive	[36]
Naucletine	*Nauclea* *officinalis*	Injection, 1 × 10^−5^ M	Rat	Phenylephrine-induced vasodilation assay	Hypotensive	[36]
Alstilobanine A, B, C	*Alstonia angustiloba*	-	Rat	Phenylephrine-induced vasodilation assay	Hypotensive	[37]
Undulifoline	*Alstonia undulifolia*	-	Rat	Phenylephrine-induced vasodilation assay	Hypotensive	[37]
Taberniacin A, B	*Tabernaemontana divaricata*	-	Rat	Phenylephrine-induced vasodilation assay	Hypotensive	[38]
Villocarine A	*Uncaria villosa*	Injection, 30 μM	Rat	Phenylephrine-induced vasodilation assay	Hypotensive	[39]
Macusine B	*Rauvolfia reflexa*	-	Cholinesterase Enzyme	Cholinesterase Inhibition assay	Anticholinesterase	[40]
Vinorine	*Rauvolfia reflexa*	-	Cholinesterase Enzyme	Cholinesterase Inhibition assay	Anticholinesterase	[40]
Isoreserpiline	*Rauvolfia reflexa*	-	Cholinesterase Enzyme	Cholinesterase Inhibition assay	Anticholinesterase	[40]
Rescinnamine	*Rauvolfia reflexa*	-	Cholinesterase Enzyme	Cholinesterase Inhibition assay	Anticholinesterase	[40]
Voacangine hydroxyindolenine	*Tabernaemontana australis*	-	Cholinesterase Enzyme	Cholinesterase Inhibition assay	Anticholinesterase	[41]
Rupicoline	*Tabernaemontana australis*	-	Cholinesterase Enzyme	Cholinesterase Inhibition assay	Anticholinesterase	[41]
Coronaridine	*Ervatamia hainanensis*	-	Cholinesterase Enzyme	Cholinesterase Inhibition assay	Anticholinesterase	[42]
Voacangine	*Ervatamia hainanensis*	-	Cholinesterase Enzyme	Cholinesterase Inhibition assay	Anticholinesterase	[42]
Angustidine	*Nauclea officinalis*	-	Cholinesterase Enzyme	Cholinesterase Inhibition assay	Anticholinesterase	[43]
Nauclefine	*Nauclea officinalis*	-	Cholinesterase Enzyme	Cholinesterase Inhibition assay	Anticholinesterase	[43]
Angustine	*Nauclea officinalis*	-	Cholinesterase Enzyme	Cholinesterase Inhibition assay	Anticholinesterase	[43]
Harmane	*Perganum harmala*	100–200 µM	Rabbit Platelet	-	Antiplatelet	[44]
Harmine	*Perganum harmala*	100–200 µM	Rabbit Platelet	-	Antiplatelet	[44]
Harmol	*Perganum harmala*	100–200 µM	Rabbit Platelet	-	Antiplatelet	[44]
Kurryam	*Murraya koenigii*	10, 30, and 50 mg/kg	Rat	Castor oil-induced test	Antidiarrheal	[45]
Koenimbine	*Murraya koenigii*	10, 30, and 50 mg/kg	Rat	Castor oil-induced test	Antidiarrheal	[45]
Bisnordihydrotoxiferine	*Strychnos trinervis*	Intraperitoneal, 3.12–25.00 mg/kg	Rat, Mice	Castor oil, Magnesium sulfate, and Arachidonic acid-induced diarrhea test	Antidiarrheal	[46]
Trinervine	*Strychnos trinervis*	-	Rat fundic strip, Guinea-pig ileum	Arachidonic acid, 5-hydroxytryptamine, Histamine, and Carbachol mediated contractions	Spasmolytic	[47]
Bisnordihydrotoxiferine	*Strychnos diuaricuns*	-	Rat uterus Guinea-pig ileum	Acetylcholine, Oxytocin-induced contraction, and Histamine-induced contractions	Spasmolytic	[48]
Normacusine B	*Strychnos atlantica*	0.1, 0.3, 1.0, and 3.0 μM	Rat aorta	Phenylephrine and Serotonin-induced contractions.	Spasmolytic	[49]
Harmine	*Perganum harmala*	1–100 μM	Guinea-Pig Trachea	Histamine, Carbachol, and KCl-induced contractions	Spasmolytic	[50]
Harmane	*Perganum harmala*	1–100 μM	Guinea-Pig Trachea	Histamine, Carbachol, and KCl-induced contractions	Spasmolytic	[50]
Harmaline	*Perganum harmala*	1–100 Μm	Guinea-Pig Trachea	Histamine, Carbachol, and KCl-induced contractions	Spasmolytic	[50]
Ramiflorine A, B	*Aspidosperma ramiflorum*	-	Parasite		Antileishmanial	[51]
Ellipticine	*Ochrosia borbonica*	0.01–10 µmol·L^−1^	Mouse fibroblast cells	Triglyceride assay	Lipid-lowering	[52]
9-methoxyellipticine	*Ochrosia borbonica*	0.01–10 µmol·L^−1^	Mouse fibroblast cells	Triglyceride assay	Lipid-lowering	[52]
Vincamine	*Vinca minor*	Oral, 20 and 30 mg/kg	Rat		Lipid-lowering	[53,54]
Coronaridine	*Tabernaemontana ternifolia*	-	Bacteria	Microplate Alamar blue assay	Antimycobacterial	[55]
Globospiramine	*Voacanga globosa*	-	Bacteria	Microplate Alamar blue assay and Low-oxygen recovery assay	Antimycobacterial	[56]
Ibogaine	*Tabernaemontana citrifolia*	-	Bacteria	Bactec radiometric methodology	Antimycobacterial	[57]
Voacangine	*Tabernaemontana citrifolia*	-	Bacteria	Bactec radiometric methodology	Antimycobacterial	[57]
Voacamine	*Tabernaemontana arborea*.	-	Bacteria	Resazurin microtiter assay	Antimycobacterial	[58]

**Table 2:** Pharmacokinetic profiles of indole alkaloids.

Parameters	Compound Name				
	Brucine	Harmane	Vindoline	Mitragynine	7-OH-mitragynine
Administration Route	Oral, IV	Oral, IV	Oral, IV	Oral	Oral
Oral Bioavailability	40.31%–47.15%	19.41%	5.4%	-	-
AUC	Ratio: 1:1.9:5.3 (at different doses)	-	Oral: 606.3 ng/mL h, IV: 2245.7 (first dose), 2258.0 (second dose) ng/mL h	-	-
C_max_	929.22–1451.58 μg/L	Oral: 1059.56 ± 91.06 ng/mL, IV: 583.19 ng/mL	Oral: 606.6 ng/mL, IV: 1595.9 (first dose), 2913.9 ng/mL (second dose)	0.63 ± 0.18 µg/mL	-
T_max_	0.3–0.5 h	Oral: 0.23 ± 0.06 h, IV: 0.03 h	Oral: 0.3 h	1.83 ± 1.25 h	-
t_1/2_	-	-	Oral: 0.5 h IV: 1.0 h (first dose), 1.4 h (second dose)	-	24 min
V_d_	-	-	Oral; 21.6 L/kg IV: 2.0 L/kg (first dose), 3.3 L/kg (second dose)	89.50 ± 30.30 L/kg	-
CL	-	-	Oral: 26.6 L/h/kg IV: 1.4 L/h/kg (first dose), 1.4 L/h/kg (second dose)	-	43.2 ± 3.5 mL/min/kg
References	[60]	[64]	[63]	[61]	[62]

## Data Availability

Available data are presented in the manuscript.
